# Comparative Assessment of the Antioxidant and Anticancer Activities of *Plicosepalus acacia* and *Plicosepalus curviflorus*: Metabolomic Profiling and In Silico Studies

**DOI:** 10.3390/antiox11071249

**Published:** 2022-06-25

**Authors:** Enas E. Eltamany, Marwa S. Goda, Mohamed S. Nafie, Abdelghafar M. Abu-Elsaoud, Rawan H. Hareeri, Mohammed M. Aldurdunji, Sameh S. Elhady, Jihan M. Badr, Nermeen A. Eltahawy

**Affiliations:** 1 Department of Pharmacognosy, Faculty of Pharmacy, Suez Canal University, Ismailia 41522, Egypt; enas_mostafa@pharm.suez.edu.eg (E.E.E.); marwa_saeed@pharm.suez.edu.eg (M.S.G.); nermeen.azmy@pharm.suez.edu.eg (N.A.E.); 2 Department of Chemistry, Faculty of Science, Suez Canal University, Ismailia 41522, Egypt; mohamed_nafie@science.suez.edu.eg; 3 Department of Botany and Microbiology, Faculty of Science, Suez Canal University, Ismailia 41522, Egypt; abuelsaoud@science.suez.edu.eg; 4 Department of Pharmacology and Toxicology, Faculty of Pharmacy, King Abdulaziz University, Jeddah 21589, Saudi Arabia; rhhareeri@kau.edu.sa; 5 Department of Clinical Pharmacy, College of Pharmacy, Umm Al-Qura University, P.O. Box 13578, Makkah 21955, Saudi Arabia; mmdurdunji@uqu.edu.sa; 6 Department of Natural Products, Faculty of Pharmacy, King Abdulaziz University, Jeddah 21589, Saudi Arabia

**Keywords:** *P. acacia*, *P. curviflorus*, antioxidant, anticancer, chemical profiling, docking studies, drug discovery, industrial development

## Abstract

This study presents a comparison between two mistletoe plants—*P. acacia* and *P. curviflorus*—regarding their total phenolic contents and antioxidant and anticancer activities. *P. curviflorus* exhibited a higher total phenolics content (340.62 ± 19.46 mg GAE/g extract), and demonstrated higher DPPH free radical scavenging activity (IC_50_ = 48.28 ± 3.41µg/mL), stronger reducing power (1.43 ± 0.54 mMol Fe^+2^/g) for ferric ions, and a greater total antioxidant capacity (41.89 ± 3.15 mg GAE/g) compared to *P. acacia*. The cytotoxic effects of *P. acacia* and *P. curviflorus* methanol extracts were examined on lung (A549), prostate (PC-3), ovarian (A2780) and breast (MDA-MB-231) cancer cells. The highest anticancer potential for the two extracts was observed on PC-3 prostate cancer cells, where *P. curviflorus* exhibited more pronounced antiproliferative activity (IC_50_ = 25.83 μg/mL) than *P. acacia* (IC_50_ = 34.12 μg/mL). In addition, both of the tested extracts arrested the cell cycle at the Pre-G1 and G1 phases, and induced apoptosis. However, *P. curviflorus* extract possessed the highest apoptotic effect, mediated by the upregulation of p53, Bax, and caspase-3, 8 and 9, and the downregulation of Bcl-2 expression. In the pursuit to link the chemical diversity of *P. curviflorus* with the exhibited bioactivities, its metabolomic profiling was achieved by the LC-ESI-TOF-MS/MS technique. This permitted the tentative identification of several phenolics—chiefly flavonoid derivatives, beside some triterpenes and sterols—in the *P. curviflorus* extract. Furthermore, all of the metabolites in *P. curviflorus* and *P. acacia* were inspected for their binding modes towards both CDK-2 and EGFR proteins using molecular docking studies in an attempt to understand the superiority of *P. curviflorus* over *P. acacia* regarding their antiproliferative effect on PC-3 cancer cells. Docking studies supported our experimental results; with all of this taken together, *P. curviflorus* could be regarded as a potential prospect for the development of chemotherapeutics for prostate cancer.

## 1. Introduction

Cancer is a serious public health issue, with increasing rates of occurrence and mortality [1]. It is ranked by the WHO as one of the major causes of death worldwide, and was responsible for 10 million deaths in 2020 [2,3,4,5,6,7]. Among women, ovarian and breast carcinomas are the most prevalent invasive and lethal malignancies [8,9,10,11]. Meanwhile, prostatic neoplasms are the most frequently diagnosed cancers in men, and are considered the third-leading cause of cancer-related deaths in the USA [7,12]. In past decades, immense advances in cancer research were achieved, especially in the provision of targeted prevention and treatments, which improved the quality of life and survival time remarkably. Nevertheless, cancer treatment remains a tremendous challenge due to its severe adverse effects, chemotherapy resistance or tumor progression and metastasis, and therefore worse prognoses [1,2,9,12]. Thus, there is an increased demand for new anticancer candidates to face these health concerns and reduce the cancer burden worldwide [5,6].

Herbal medicines and natural products are gaining attention due to their potential to cure stubborn diseases, including cancer [13,14]. Herbal medicines can potentiate the activity and diminish the adverse effects and organ toxicities of chemotherapy. Moreover, medicinal plants themselves and natural product-derived compounds can target cancer cells effectively and selectively, without affecting normal cells [1,15]. This might be correlated to their diverse chemotypes and pharmacological effects [14].

Phenolics are the most abundant plant secondary metabolites [16]. Both monophenolic and polyphenolic constituents from numerous plants have been reported to halt the initiation, proliferation and spread of malignant cells in vitro and in vivo. The anti-neoplastic effects are mainly attributed to the ability of phenolics to modulate ROS levels, induce cell cycle arrest, and attenuate cell proliferation, angiogenesis and apoptosis via the inhibition of oncogenic signaling cascades and the activation of tumor suppressor proteins such as p53 [17,18].

Mistletoes are hemi-parasitic woody shrubs belonging to the subclass Rosidae and the order Santalales, encompassing both the Viscaceae and Lorantheacae [19]. The Loranthaceae include approximately 70 genera and 800 species worldwide [20]. Mistletoes are used as ethnomedicinal plants for the alleviation and treatment of numerous diseases [21], including diabetes [22,23]. Furthermore, evidence of their antitumor activity has been acquired [24].

*Plicosepalus curviflorus* (Benth. ex Oliv.) Tiegh., and *Plicosepalus acacia* are two mistletoes of the family Loranthaceae which are vastly distributed in Saudi Arabia, and are commonly employed in traditional medicinal practices [25,26]. *P. curviflorus* has been utilized to cure diabetes, pneumonia and cancer, and as a galactagogue for cattle [27]. Moreover, the antimicrobial effect of *P. curviflorus* and its cytotoxicity on FL-cells (a human amniotic epithelial cell line) have been reported [28]. On the other hand, *P. acacia* was verified to have antimicrobial potential [29], multi-organ protective effects [30,31,32], anti-diabetic activity [33] and an angiogenic effect against diabetes-induced hind ischemia [34]. The exhibited bioactivities of *P. curviflorus* and *P. acacia* are attributed to their phytoconstituents. Chemical investigations of *P. curviflorus* resulted in the isolation and identification of triterpenes, phytosterols and polyphenolics: predominantly flavonols and flavan-3-ols [35,36,37,38,39,40]. Similarly, phenolic acids, flavonoids were reported in *P. acacia* in addition to loranthin a flavanocoumarin [26,34]. Interestingly, despite *P. acacia* and *P. curviflorus* are good sources of phenolic compounds, there are few scientific reports on the anticancer activity of these plants [36].

In the light of the aforementioned consideration, this study involves a comparison between *P. acacia* and *P. curviflorus* with respect to their contents of phenolic compounds, antioxidant potentials and most importantly anticancer activities. The metabolomic profiling of *P. curviflorus* was achieved using the LC-MS/MS technique. Besides this, the molecular docking tool was employed to determine the most bioactive compounds by exploring their binding affinities towards Cyclin-dependent kinase-2 (CDK-2) and epidermal growth factor receptor (EGFR) proteins.

## 2. Materials and Methods

### 2.1. Preparation of the Extracts

In March 2010, *P. acacia* and *P. curviflorus* were previously collected from the Saudi Arabian cities of Al-ula (in the Madina region) and Abha (in the Asir region), respectively [34,38]. The plants were authenticated in the Faculty of Science, King Abdulaziz University, Jeddah, Saudi Arabia by Dr. Nahed Morad. Voucher samples under the registration codes 2010-PC1 and 2010-PA1 for *P. curviflorus* and *P. acacia*, respectively, were placed at the Department of Natural Products, Faculty of Pharmacy, King Abdulaziz University. The plant samples were then air dried and ground. The extracts of *P. curviflorus* and *P. acacia* were prepared by macerating 1.5 Kg of each plant with MeOH (3 L, twice) at ambient temperature. Then, the extracts were concentrated under reduced pressure and stored in a refrigerator.

### 2.2. Estimation of the Total Phenolic Content

The total phenolics in the *P. acacia* and *P. curviflorus* extracts were quantified spectrophotometrically by the Folin–Ciocalteu method, as described previously in [41]. In brief, a 200 µg/mL methanolic solution of the plant sample was prepared. The test solution (0.5 mL) was mixed with Folin–Ciocalteu reagent (2.5 mL). Then, 2 mL Na_2_CO_3_ solution with a concentration of 75 mg/mL was added. The reaction mixture was kept for 10 min at 50 °C. Against the blank, the UV absorbance was recorded at λ 630 nm using a Milton Roy, Spectronic 1201 (Houston, TX, USA) and gallic acid as a standard. The result was expressed in terms of gallic acid equivalents (mg·GAE/g dry extract).

### 2.3. Evaluation of the In Vitro Antioxidant Activity

#### 2.3.1. DPPH Free Radical Scavenging Activity

*P. acacia* and *P. curviflorus* extracts were investigated for their free radical-scavenging activities by applying the method mentioned in [42,43]. Briefly, a solution of the free radical—2,2-diphenyl-1-picrylhydrazyl (DPPH)—in methanol was freshly prepared with a concentration of 0.004% *w*/*v*, then kept in dark at 10 °C. Various concentrations of the tested extract solution were prepared. Then, the test solution (40 µL) was added to the DPPH solution (3 mL). The mixture was incubated at ambient temperature for 30 min, in the dark. After this, the absorbance was measured at λ 515 nm against a blank using a UV/Vis spectrophotometer (Milton Roy, Spectronic 1201, Houston, TX, USA). In addition, the absorbance of ascorbic acid (the standard) was recorded. The absorbances of the reaction mixtures were recorded in triplicate. The inhibition % (PI) of the DPPH radical was derived from this equation:

PI = [{(AC − AT)/AC} × 100]

AC is the control absorbance and AT is the absorbance of DPPH + sample. The 50% inhibitory concentration (IC_50_) was obtained from the dose/response curve constructed by Graphpad Prism 7 software (Dotmatics, San Diego, CA, USA).

#### 2.3.2. Ferric Reducing Antioxidant Power (FRAP) Assay

The FRAP of the *P. Acacia* and *P. curviflorus* extracts were estimated spectrophotometrically using the procedure described [44,45]. This procedure was based on ferricyanide ion reduction in proportion to different concentrations of the tested sample. Briefly, the methanolic solution of the extract (1 mL) was added to 0.2 M sodium phosphate buffer with pH = 6.6 (2.5 mL) and 1% *w*/*v* potassium ferricyanide solution (2.5 mL). The mixture was kept for 20 min at 50 °C, then acidified with 10% *w*/*v* trichloroacetic acid (2.5 mL), and then centrifuged at 650 rpm for 10 min. After that, the supernatant (2.5 mL) was mixed with deionized water (2.5 mL) and 0.1% *w*/*v* freshly prepared ferric chloride (0.5 mL). Then, the absorbance of the reaction mixture was recorded at λ 700 nm against a blank using a UV/Vis spectrophotometer (Milton Roy, Spectronic 1201, Houston, TX, USA). Butyl hydroxy toluene (BHT) was employed as a standard. The obtained results were expressed in terms of m Mol Fe^+2^ equivalent/g dry sample.

#### 2.3.3. Total Antioxidant Capacity (TAC) Assay

The *P. acacia* and *P. curviflorus* extracts’ total antioxidant capacities (TAC) were estimated spectrophotometrically using a phosphomolybdenum assay. The procedure was executed according to [46]. This assay is based on the capability of an antioxidant substance to reduce Mo (VI) to Mo (V) in an acidic medium, producing a phosphate/Mo (V) complex with a green color. In this test, 0.2 mL of the methanolic solution of the extract was mixed with to 1 mL of the reagent solution composed of 2 mM sodium phosphate, 4 mM ammonium molybdate and 0.6 M sulphuric acid. The reaction mixture was then incubated at 95 °C for 90 min, and was then cooled. Finally, the absorbance of the mixture was read at λ 695 nm using a UV/Vis spectrophotometer (Milton Roy, Spectronic 1201, Houston, TX, USA). Ascorbic acid served as a standard. The data were expressed as mg equivalents of gallic per g extract (mg GAE/g) using the standard curve of gallic acid.

### 2.4. Anticancer Activity of P. acacia and P. curviflorus

#### 2.4.1. Cytotoxic Activity

Prostate (PC-3), lung (A549), breast (MDA-MB-231) and ovarian (A2780) cancer cell lines were purchased from the National Cancer Institute, Cairo, Egypt. The cells were kept in Dulbecco’s Modified Eagle Medium (DMEM, Sigma-Aldrich, St. Louis, MO, USA) supplemented with 10% fetal bovine serum (FBS, Sigma-Aldrich, St. Louis, MO, USA), 2 mM L-glutamine (Lonza, Belgium) and 1% penicillin-streptomycin (Lonza, Belgium). After this, 5 × 10^3^ cells were placed in a 96-well microplate (in triplicates) and left for 48 h. Then, the cells were treated with *P. acaciae* and *P. curviflorus* extracts, at the concentrations of 0.1, 1, 10, and 100 µg/mL, and were incubated for 48 h. For the assessment of the cell viability, 20 μL MTT dye (Promega, Madison, WI, USA) (Mosmann, 1983) was transferred to the wells and the plate was kept for 3 h. Using an ELISA microplate reader (BIO-RAD, model iMark, Osaka, Japan), the absorbance was subsequently recorded (at λ 570 nm). As was previously reported by [47,48], the cell viability was estimated with respect to a control and the IC_50_ values were obtained using GraphPad prism 7.

#### 2.4.2. Annexin V/PI Staining and Cell Cycle Analysis

The rate of apoptosis in PC-3 cells was estimated by means of annexin V-FITC (BD Pharmingen, San Diego, CA, USA). The cells were added into 6-well culture plates with a concentration of 3–5 × 10^5^ cells/well and left to incubate overnight. *P. acaciae* and *P. curviflorus* extracts were added at their IC_50_ values to the cultured cells, and were incubated for 48 h. After that, the cells and media supernatants were collected and washed with ice-cold PBS. The cells were then suspended in 100 µL annexin binding buffer solution composed of 1.4 M NaCl, 25 mM CaCl_2_, and 0.1 M Hepes/NaOH, and the pH was adjusted to 7.4 and incubated in the dark for 30 min with annexin V-FITC solution (1:100) and propidium iodide (PI) at a concentration equal to 10 µg/mL. The Cytoflex FACS machine (Beckman Coulter Inc., Brea, California, USA) was used to acquire the stained cells. CytExpert software (V2.4, Beckman Coulter Inc., California, USA)was utilized for the data evaluation [49,50].

#### 2.4.3. RT-PCR for the Apoptosis-Related Genes

For the further inspection of the apoptotic pathway, we tracked the expression of the pro-apoptotic genes (P53, Bax, and Caspapses-3,8,9) and the anti-apoptotic gene Bcl-2. Table 1 summarizes their sequences in forward and reverse directions.

PC-3 cells were treated with *P. curviflorus* extract at its IC_50_ value, then incubated for 48 h. Then, RNA extraction, cDNA synthesis, and RT-PCR reaction were performed. All of the reactions were accomplished for 35 cycles at 95 °C for 5 min (initial denaturation); 95 °C for 15 min (Denaturation), 55 °C for 30 min (Annealing), and 72 °C for 30 min (Extension). Finally, the Ct values were obtained in all of the samples, and were employed in the estimation of the relative genes’ expression. β-actin, the housekeeping gene, was used as a normal control [51,52,53].

### 2.5. Metabolomic Profiling by LC/MS/MS

High-resolution LC-ESI-TOF-tandem mass spectrometic analysis was executed as previously mentioned in [34,41]. *P. curviflorus* extract (50 mg) was dissolved in 1 mL of the mobile phase composed of water:methanol:acetonitrile (50:25:25). The solution was sonicated (10 min) and then centrifuged at 10,000 rpm (10 min). An aliquot (50 µL) of the prepared solution was withdrawn and further diluted with the reconstitution solvent. Finally, 2.5 µg/µL of the solution was prepared, of which 10 µL was injected in both the negative and positive modes. For confidence assurance in our experiment, blanks were also analyzed. For the positive mode, the mobile phase constituted of 5 mM ammonium formate buffer in 1% methanol (pH = 3.0) was employed. On the other hand, the pH of the aforementioned buffer was adjusted to 8.0 to suit the negative mode. The UHPLC separation was achieved using an ExionLC system (AB Sciex, Framingham, MA, USA) with a 2.5 µm, 2.1 × 150 mm X select HSS T3 column (Waters Corporation, Milford, MA, USA), Phenomenex^®^ in-line filter disks (0.5 µm × 3.0 mm), and an autosampler system; the flow rate was 0.3 mL/min. For the MS/MS fragmentation spectra, this compartment was attached to a Triple TOF™ 5600+ system (AB SCIEX, Concord, NC, Canada). MS-DIAL3.52 was utilized for the data processing. Master view software was employed for the peak extraction from the total ion chromatogram (TIC) according to the criteria reported previously by Eltamany and coworkers [41]. The compounds were identified by accurate mass estimations, MS/MS transitions, and the comparison of their retention times to those reported in the literature and mass spectral databases for LC/MS-based metabolomic analysis.

### 2.6. Statistical Analyses

Using Microsoft Excel 2016, the obtained data were collected and depicted in tables and figures. The data were subjected to outlier detections and normality statistical tests in order to detect whether the data were parametric or nonparametric using a Shapiro-Wilk normality test at the 0.05 level. The data were described statistically in terms of means and standard deviations. Inferential statistics for evaluating and comparing between *P. curviflorus* and *P. acacia* were produced using independent sample *t*-tests including ascorbic acid and BHT using one way analysis of variance at the 0.05 level. ANOVA was followed by Duncan’s Multiple Range test (DMRTs) to further compare the groups. Data analyses were carried out using the computer software Statistical Package for Social Science (SPSS) (IBM-SPSS ver. 28.0 for Mac OS). PCA ordination was performed using PAST (Paleontological Statistics) statistical software (Oslo, Norway) version 4.09 for Mac OS.

### 2.7. Molecular Docking

Computational docking experiments were performed in order to investigate the interaction between *P. acaciae* and *P. curviflorus* phytochemicals and the active sites of cyclin-dependent kinase-2 (CDK-2) and epidermal growth factor receptor (EGFR). From the PDB, the target proteins (PDB:2a4l and PDB: 1M17) were unrestrictedly obtainable, the amino acids were adjusted with missing atoms or alternative positions for structure optimization. By applying Maestro, the ligand structures were constructed, optimized, and energetically stabilized. In order to perform our molecular docking study, the appropriate formats of receptors and ligands were prepared, the grid box dimensions box of 10 Å in the x, y and z directions centered on the ligand was determined, and finally docking with binding affinities in terms of ligand–receptor interactions and binding energies was investigated according to the routine work discussed by Nafie et al., 2019 [54]. In order to validate the molecular docking calculations, MOE 2019 was employed. In order to visualize and evaluate the drug–target interactions, Chimera software was used.

## 3. Results and Discussion

### 3.1. Total Phenolic Content (TPC)

**Figure 1:**
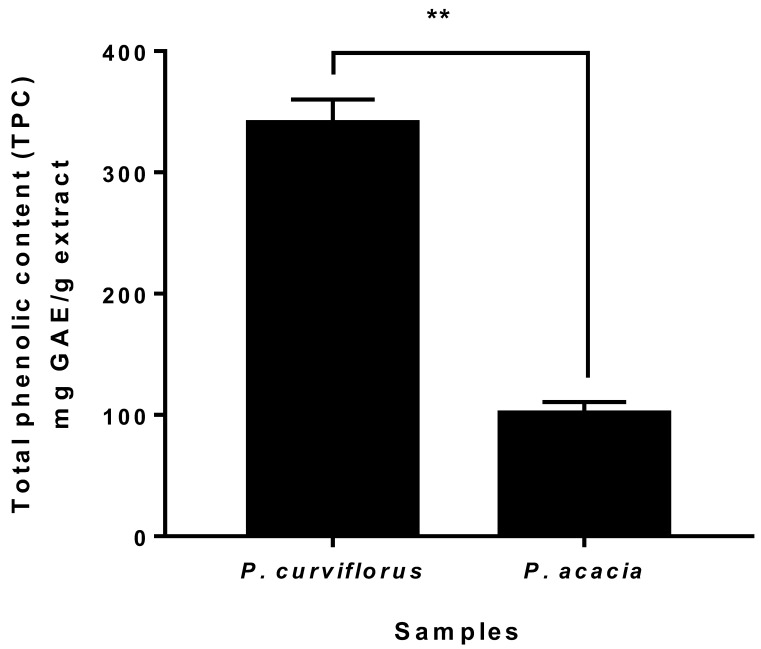
Total phenolic content (TPC) in *P. curviflorus* and *P. acacia*. The values are expressed as the mean ± SD of three independent trials. ** (*p* ≤ 0.001) Highly significant using an unpaired *t*-test.

Phenolic compounds are classified into flavonoids and non-flavonoids, which comprise stilbenes, tannins, coumarins and phenolic acids. They are the most abundant plant phytochemicals [16]. Plant polyphenols have gained great attention for their potent antioxidant effects and their preventive properties against various oxidative stress-associated diseases, particularly cancer [55]. Consequently, the estimation of the total phenolics in plant extracts is rational for the determination of their antioxidant power. The total phenolic contents (TPC) of *P. curviflorus* and *P. acacia* extracts were appraised by the Folin–Ciocalteu colourimetric method. Derived from the gallic acid calibration curve, the linear equation obtained was Y = 0.0011X + 0.0131 with a coefficient of determination R^2^ = 0.9946. The total phenolic contents of the *P. curviflorus* and *P. acacia* methanolic extracts were 340.62 ± 19.46 mg GAE/g extract and 101.15 ± 9.53 mg GAE/g extract, respectively. Figure 1 demonstrates the statistically highly significant difference (*p* < 0.001) between both species in TPC, as revealed by an independent sample *t*-test.

### 3.2. Evaluation of the In Vitro Antioxidant Activity of P. curviflorus and P. acacia

Phenolics have one or more aromatic benzene rings with mono- or poly-hydroxyl groups in their structure [16]. Thus, they possess electron- or hydrogen-donation properties and reducing and metal-chelating capabilities [41]. Several research papers have evidenced a positive correlation between oxidative stress and the progression of serious health problems. In this sense, antioxidants can diminish this stress, resulting in disease prevention. Owing to the various ROS (reactive oxygen species) scavenging modes and the complexity of natural products, a group of assays was employed concurrently for the evaluation of the antioxidant activities of plant extracts. Herein, three indicative assays (DPPH, FRAP, TAC) were used to inspect and compare the antioxidant power of *P. curviflorus* and *P. acacia* extracts. The results are depicted in Table 2.

Our findings indicated that *P. curviflorus* and *P. acacia* extracts varied in their capacities to scavenge DPPH free radicals. The calculated IC_50_ of the DPPH free radical scavenging of *P. curviflorus* and *P. acacia* were 48.28 ± 3.41 µg/mL and 60.70 ± 4.28 µg/mL, respectively, compared to ascorbic acid as a standard (IC_50_ 10.64 ± 0.82 µg/mL). These results revealed that *P. curviflorus* extract demonstrated better DPPH free radical neutralizing activity with a lower (IC_50_) value compared to *P. acacia*. A one-way ANOVA test proved the presence of a highly significant difference between the tested extracts. Moreover, statistical means accompanied by different letters are significantly different according to Duncan’s Multiple Range Test at the 0.05 level.

These findings could be linked to the differences in TPC between *P. curviflorus* and *P. acacia* extracts, as several studies have evidenced the strong association between the antioxidant activity estimated by DPPH assay and TPC and TFC levels, principally owing to the phenolic compounds’ redox properties [56]. Thus, they can donate an electron or hydrogen radical to a DPPH free radical, transforming it into a neutralized stable diamagnetic molecule [57].

The results from the FRAP assay indicated that both of the tested extracts had notable Fe^+3^ reduction activity. *P. curviflorus* extract exhibited a non-significantly (*p* > 0.05) stronger Fe^+3^ reducing power (1.43 ± 0.54 mMol Fe^+2^/g) than *P. acacia* (1.29 ± 0.31 mMol Fe^+2^/g), and the Fe^+3^ reducing power of the positive controls BHT and ascorbic acid were 8.07 ± 0.79 mMol Fe^+2^/g and 3.14 ± 0.82 mMol Fe^+2^/g, respectively. The stronger Fe^+3^ reducing potential of *P. curviflorus* is also in line with its higher TPC. Like DPPH, the obtained results from the FRAP assay demonstrated that *P. curviflorus* extract has a comparatively better depository of antioxidants than *P. acacia* extract. The FRAP assay results revealed a statistically significant difference using one-way ANOVA. Moreover, means followed by different letters are significantly different according to Duncan’s Multiple Range Test at the 0.05 level.

The phosphomolybdate test is another antioxidant assay that estimates the capability of a sample to obliterate a free radical by electron-transferring to the later. Data has shown that *P. curviflorus* (41.89 ± 3.15 mg GAE/g) exhibited a higher TAC than *P. acacia* (32.67 ± 2.81 mg GAE/g), while the TAC of ascorbic acid (the positive control) was 69.23 ± 4.51 mg GAE/g. Evidence of the antioxidant and antitumor effects of polyphenolics has been acquired in several studies, and is well accepted [5,57,58,59,60,61,62,63,64,65]. Thus, the exhibited variation in the total antioxidant capacity between *P. curviflorus* and *P. acacia* may be attributed to the differences in their total phenolic contents.

### 3.3. Evaluation of the Anticancer Activity of P. acacia and P. curviflorus

#### 3.3.1. Cytotoxicity Using the MTT Assay

**Figure 2:**
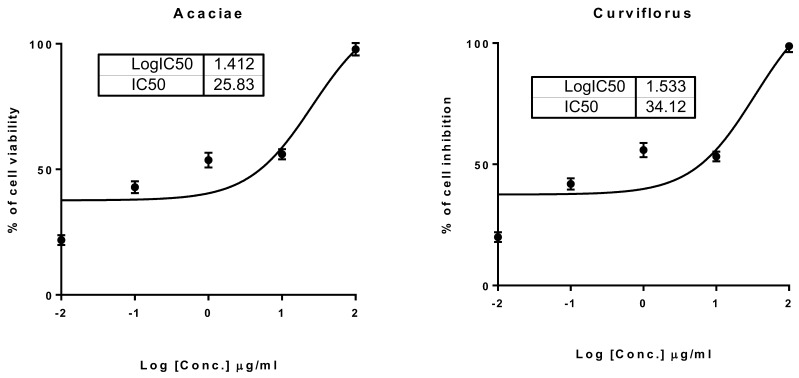
The constructed dose–response nonlinear regression curve fitting the percentage of cell viability vs. log [con. µg/mL], R square *≈* 1, using GraphPad prism software. A, Cytotoxicity of *P. acaciae* against prostate cancer PC-3 cells; B, cytotoxicity of *P. curviflorus* against prostate PC-3 cells. The incubation time for the cell lines with the treatments was 48 h.

The extracts of *P. acacia* and *P. curviflorus* were tested for their cytotoxicity against a panel of cell lines using an MTT assay. As depicted in Table 3, both of the extracts exhibited promising cytotoxic activities against PC-3 cells, with IC_50_ values of 34.12 and 25.83 μg/mL, respectively. Both extracts caused high percentages of inhibition of PC-3 cell proliferation of around 98%, but exhibited poor cytotoxicity against other cell lines (Figure 2).

#### 3.3.2. Apoptosis-Inducing Activity

**Figure 3:**
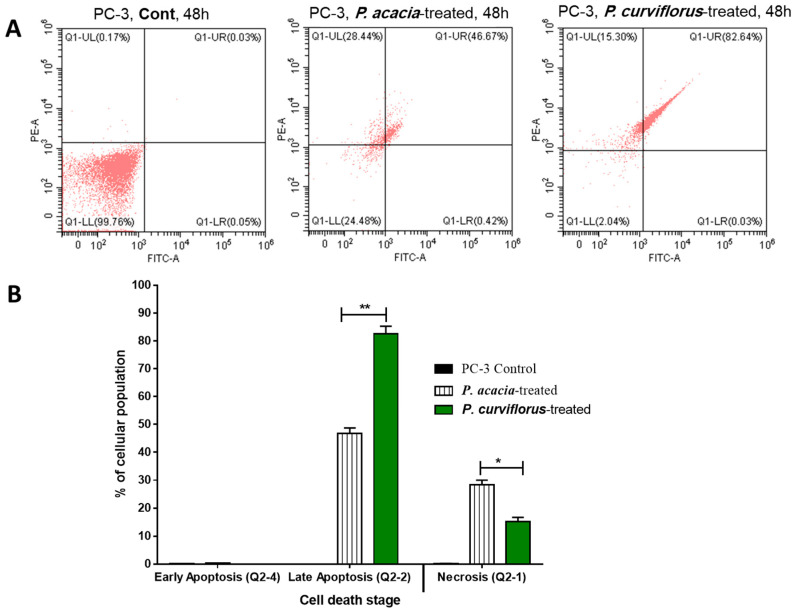
(**A**) FITC/Annexin-V-FITC/PI profiling of the apoptosis/necrosis of PC-3 cells treated with the extracts of *P. acaciae* (IC_50_ of 34.12 µg/mL, 48 h) and *P. curviflorus* (IC_50_ of 25.83 µg/mL, 48 h), and the untreated controls. Q-UL: necrosis, AV−/PI+; Q-UR: late apoptotic cells, AV+/PI+; Q-LL: normal cells, AV−/PI−; Q-LR: early apoptotic cells, AV+/PI−. (**B**) Bar representation for the percentage of apoptotic and necrotic cell death in the untreated and treated PC-3 cells. * *p* ≤ 0.05 and ** *p* ≤ 0.001 are significantly different between treated and untreated cells using an unpaired *t*-test in GraphPad prism.

**Figure 4:**
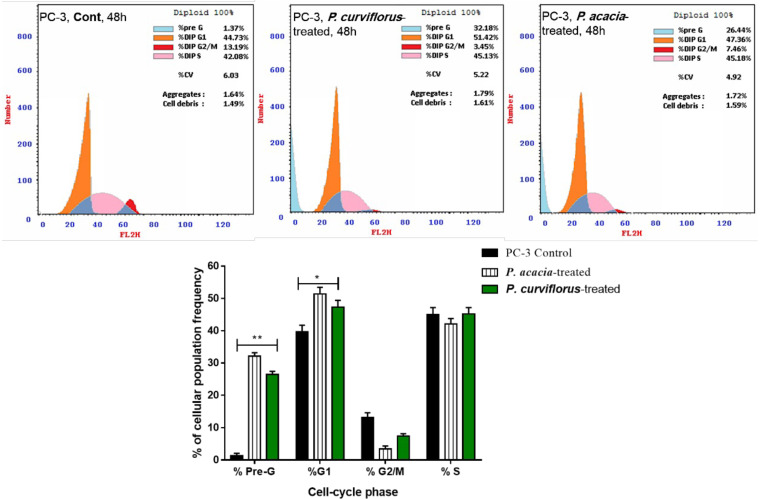
Cell cycle analysis of the untreated and treated PC-3 cells with both extracts of *P. acaciae* (IC_50_ of 34.12 µg/mL, 48 h) and *P. curviflorus* (IC_50_ of 25.83 µg/mL, 48 h). The values are expressed as the mean ± SD of three independent experiments. * *p* ≤ 0.05 and ** *p* ≤ 0.001 are significantly different between treated and untreated cells using an unpaired *t*-test in GraphPad prism.

The *P. curviflorus* and *P. acacia* extracts were tested for apoptosis-inducing activities in PC-3 cells at their IC_50_ values. The results are demonstrated in Figure 3A,B. Interestingly, *P. curviflorus* extract exhibited promising apoptotic cell death by 82.67% compared to 0.08% in the untreated control, mainly with 82.64% as late apoptosis, while it caused necrosis by 15.30%, compared to 0.17 in the untreated control. Additionally, *P. acacia* extract exhibited apoptotic cell death by 47.09%, compared to 0.08% in the untreated control, mainly as 46.7% as late apoptosis, while it caused 28.44% compared to 0.17 in the untreated control. Hence, the results showed promising apoptotic activity over necrosis. After the treatment of PC-3 cells with both extracts as cytotoxic agents, cell cycle analysis was performed. As seen in Figure 4, both of the extracts significantly increased the cell population in the G1-phase in comparison with the control cells (untreated), while they decreased the cell population in both the G2/M and S-phases. Upon treatment with both extracts, the cells were accumulated in the pre-G1 phase, as evidenced by the existence of a sub-G1 peak when analyzing the cell cycle profile. This may have been a result of genetic material degradation or fragmentation, suggesting the incidence of apoptosis. Hence, both extracts triggered cell cycle arrest in the pre-G1 and G1-phases following cytotoxically induced activities in PC-3 cells.

#### 3.3.3. RT-PCR Apoptosis-Related Genes

**Figure 5:**
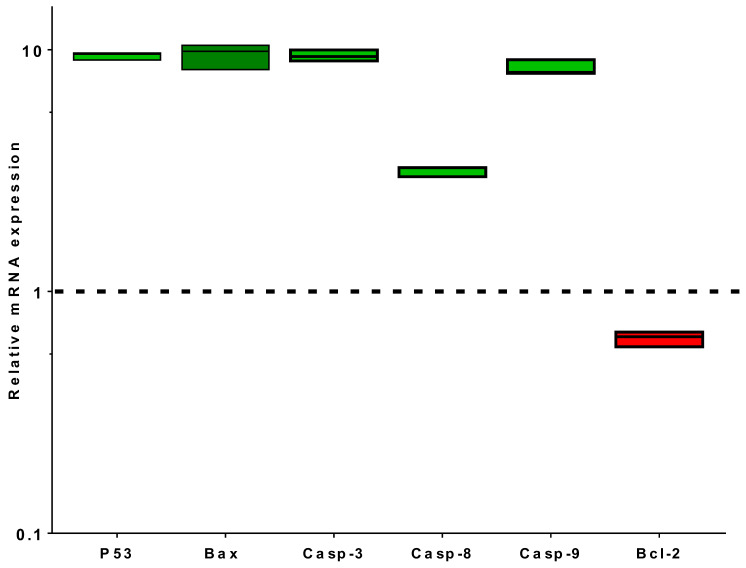
Analysis of the mRNA gene expression of PC-3 cells treated with *P. curviflorus extracts* (IC_50_ of 25.83 µg/mL, 48 h) and the untreated cells. The fold of change of the untreated control = 1. The values are expressed as the mean ± SD of three independent values.

The promising apoptotic effect of *P. curviflorus* extract in PC-3 cells was further scrutinized through the profiling the expression of apoptosis-regulating genes—caspase-3-8-9, P53, Bax and Bcl-2—in treated and untreated PC-3 cells. *P. curviflorus* extract treatment induced the upregulation of the expression of p53 9.5-fold, Bax 9.85-fold, caspase-3 9.38-fold, caspase-8 3.25-fold, and capsae-9 8.06-fold, while Bcl-2 expression was downregulated 0.65-fold. Our results suggest apoptosis induction in PC-3 via pro-apoptotic gene upregulation and anti-apoptotic gene downregulation. P53 is a tumor suppressor gene that triggers many target genes. Caspase-3 and caspase-9 activation, along with Bcl-2 gene decreased expression, may cause p53 apoptosis. Furthermore, the extrinsic and intrinsic apoptotic pathways were promoted by the enhanced levels of caspase-8, caspase-3 and caspase-9 in the extract-treated cells. The results are shown in Figure 5.

### 3.4. Principle Component Analysis (PCA)

**Figure 6:**
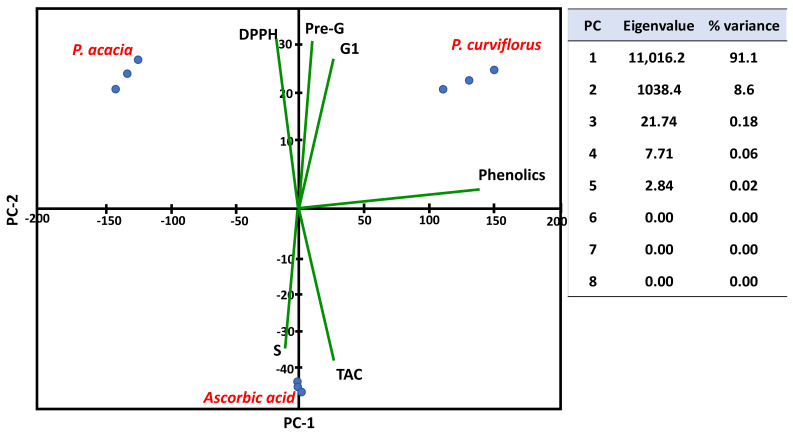
PCA-ordination based on the biochemical data of *P. acacia* and *P. curviflorus*.

**Figure 7:**
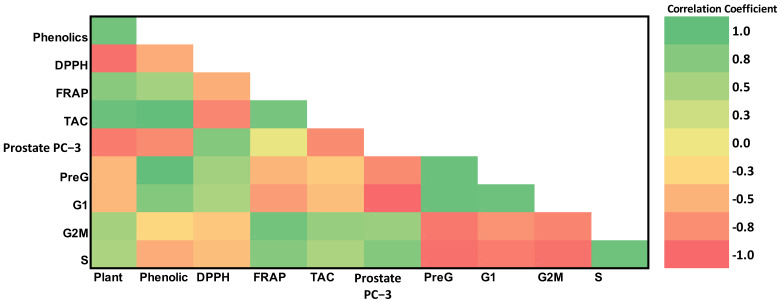
Heat map presenting the interactions and interrelationships between the study variables.

Various measured variables of *P. acacia* and *P. curviflorus* in all of the samples of the study were plotted in relation to the first and second principal components (PC-1 and PC-2). PC-1 and PC-2 represented more than 99% of the total variance, as presented in Figure 6. These show that there is a distinctive feature between *P. acacia* and *P. curviflorus* according to various estimated variables, including total phenolic compounds, TAC, and DPPH, etc. Moreover, the interrelationships between various parameters are presented as a heat map (Figure 7).

### 3.5. LC-ESI-TOF-MS/MS Analysis of P. curviflorus

**Figure 8:**
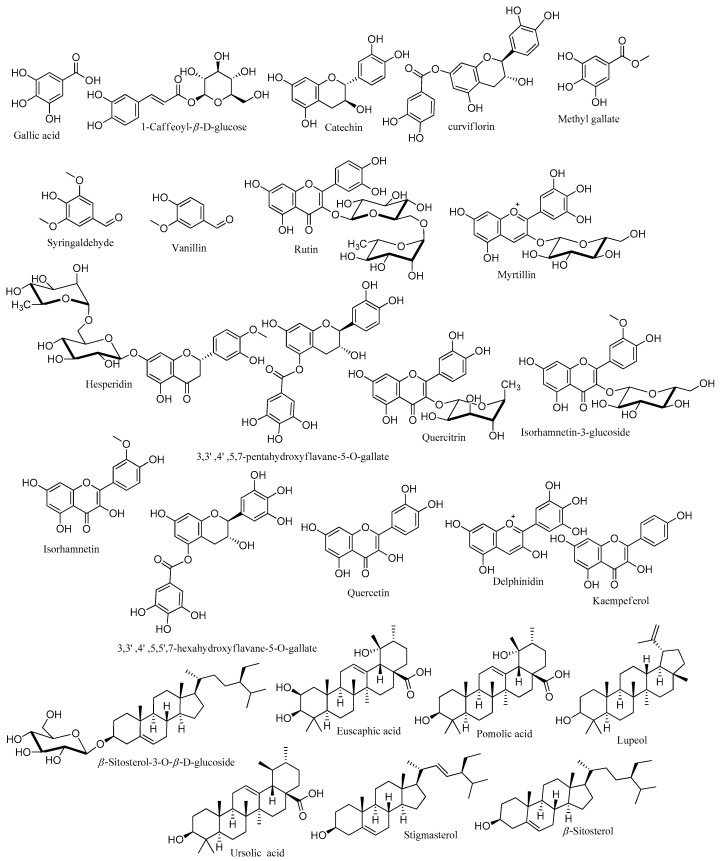
Chemical structures of the identified compounds given by LC-ESI-TOF-MS/MS.3.6. Molecular docking studies.

In the present study, *P. curviflorus* extract was verified to be a rich source of phenolic compounds, with a TPC of 340.62 ± 19.46 mg GAE/g. It exhibited a remarkable antioxidant effect, with IC_50_ = 48.28 ± 3.41 µg/mL for DPPH free radicals, an FRAP of 1.43 ± 0.54 mMol Fe^+2^/g, and a TAC of 41.89 ± 3.15 mg GAE/g. In addition, *P. curviflorus* demonstrated an auspicious anticancer effect against the PC-3 prostate cancer cell line (IC_50_ = 25.83) via the induction of apoptosis. Therefore, the metabolomic profiling of *P. curviflorus* extract was performed by the LC-ESI-TOF-MS/MS technique (Figures S1 and S2) in order to inspect the chemical diversity of its metabolites, particularly the phenolics and terpenoids responsible for the exhibited antioxidant and anticancer activities of the plant. The individual components were identified tentatively by comparing the precursors’ *m*/*z* values, MS/MS fragments and retention times with those cited in the literature. Herein, 25 hits were identified in the *P. curviflorus* extract, among which phenolics predominated (Table 4, Figure 8). Two anthocyanins (myrtillin and delphinidin) were detected for the first time in this plant. All of the identified flavan-3-ols (catechins) were isolated previously from *P. curviflorus* [27,35,36,38,39,40]. Six flavonols were recorded, of which quercetin has been reported previously in *P. curviflorus* [35,36,38,39,40], while rutin, quercitrin, kaempferol, isorhamnetin-3-glucoside and isorhamnetin were reported for the first time in this plant. Nevertheless, these were not identified in our extract; instead, their aglycone was detected. All of the detected terpenes and sterols in the present study were reported in *P. curviflorus* [35,36,38], except for euscaphic acid and stigmasterol. Gallic acid and its methyl ester, along with 1-Caffeoyl-*β*-D-glucose, vanillin and Syringaldehyde were recorded. Earlier studies have reported the isolation of gallic acid and 1-Caffeoyl-*β*-D-glucose from this plant [38,40]. However, other reported phenolic acids [40]—such as chlorogenic, caffeic and 4-methoxycinnamic acid—were not identified.

Regarding the exhibited antioxidant and anticancer activities, *P. curviflorus* extract owes such biological effects to its phytoconstituents. Phenolic compounds are well known for their antioxidant potential and their preventive effects against various oxidative stress-associated diseases, particularly cancer, via the modulation of carcinogenesis. Numerous in vitro and in vivo models have been employed to inspect the anticarcinogenic and anticancer effects of natural phenolic compounds [55]. Delphinidin was reported to prompt apoptosis in prostate cancer PC-3 Cells by interfering with nuclear factor-κB signaling [66,67]. Moreover, in human prostate cancer LNCaP cells, delphinidin triggers caspase- and p53-mediated apoptosis. Meanwhile, evidence has been acquired of the inhibitory effect of myrtillin (delphinidin-3-glucoside) on dihydrotestosterone (DHT)-induced cell growth, which is associated with decreased prostate-specific antigen production [67]. Delphinidin-3-glucoside was also reported to attenuate breast cancer progression by Akt/HOTAIR signaling pathway deactivation [68]. In earlier studies, the antimutagenic, antitumor and cancer-preventive effects of catechins (flavan-3-ols) have been proven [69]. For example, catechin extract from tea leaves was reported to inhibit PC-3 proliferation, arrest their cycle at the S phase via the elevation of p27 expression and the attenuation of cyclin A, cyclin B and consequently CDK2, and CDK1 expressions, and finally the induction of apoptosis through caspase-dependent and -independent pathways [70]. Furthermore, (+) catechin was proven to possess an in vitro antiproliferative effect against the A549 lung cancer cell line by the inhibition of cyclin E1 and P–AKT and the induction of p21, a potent CKI (cyclin kinase inhibitor) [71]. In addition, Thomas and Dong proved the apoptosis-inducing effect of (-) epicatechin on breast and prostate carcinomas, mediated by its binding to ZIP9 [72]. Besides this, 3,3’,4,5,7-pentahydroxyflavane-5-*O*-gallate exhibited promising in vitro anticancer activity against HCT-116 and HeLa cancerous cells [36]. Flavonoids have been demonstrated to reduce cancer cells’ viability by cell cycle arrest and the induction of apoptosis [73]. For instance, quercetin intake has been associated with the cell cycle arrest of PC-3 in the G0/G1 phase as a consequence of decreasing cyclin E, cyclin D, and CDK2 levels [74,75]. Moreover, the combination of quercetin with epigallocatechin gallate prompted apoptosis in LNCaP and PC3 cells through the upregulation of the p53 tumor suppressor gene [65]. The antiproliferative effect of hesperidin on HeLa, MCF-7-GFP-Tubulin and LNCaP cancer cell lines was proven [76,77]. Furthermore, Da and coworkers demonstrated the apoptotic effect of kaempferol mediated by the androgen-dependent pathway and its vasculogenic mimicry and invasion suppression in prostate cancerous cells [78]. Quercitrin was proven to significantly inhibit both DLD-1 and NSCLC cancer cells’ proliferation via the induction of apoptosis mediated by the activation of caspase-3 and the loss of mitochondrial membrane potential. Moreover, this flavonoid induced apoptosis in SGC790 gastric cancer cells through the reduction of the phosphorylated PI3K/AKT levels, resulting in the inhibition of one of the vital signaling transduction pathway in cancers [79]. In a recent study, the in vitro and in vivo antitumor activity of isorhamnetin against human gallbladder cancer cells (GBC) was evidenced. This compound induced cell cycle arrest in the G2/M phase, downregulated the expression of CDK1 and cyclin B1, and upregulated the expression of p53, CDK inhibitor and p27, and deactivated the PI3K/AKT signaling pathway, thereby halting metastasis [80]. Another study has reported the antiproliferative and antimetastatic effects of isorhamnetin towards DU145 and PC3—androgen-independent prostate cancerous cells—through the inhibition of PI3K/AKT/mTOR signaling and the activation of mitochondrion-dependent intrinsic apoptotic pathways [81]. In 2019, the Satari research team delineated the synergetic apoptotic effect of rutin-5-fluorouracil in PC-3, mediated by the upregulation of the p53 tumor suppressor gene [82]. Additionally, phytosterols have been reported to promote apoptosis [83]. For instance, the apoptotic effect of *β*-sitosterol was observed in LNCaP and PC-3 prostate cancer cells [84,85]. Besides this, it induced p53 activation and ROS-mediated mitochondrial dysregulation in A549 cells [86]. *β*-sitosterol glucoside demonstrated antitumor activity on EAC bearing mice. The apoptogenic mechanism was mediated through the upregulation of the apoptotic genes p53 and p21, together with the activation of caspases 3 and 9 [87]. Another example is stigmasterol, which simultaneously sparked apoptosis in MGC-803 and SGC-7901 gastric cancer cells through AKT/mTOR pathway arrest [88]. Several reports concerned with the anticancer potential of triterpenes have been published. An early study demonstrated that ursolic acid has evoked apoptosis in PC-3 prostate cancer cells via both extrinsic and intrinsic pathways; besides this, it has confined cell invasion through AKT pathway inhibition [89]. Moreover, it has shown to induce apoptosis through Bcl-2 phosphorylation and degradation, and consequently the activation of caspase 9, both in LNCaP-AI and DU145 prostate cancer cell lines [90]. Besides this, ursolic acid was found to attenuate metastasis in vitro and in vivo through the downregulation of CXCR4 expression in prostate cancer models [91]. Finally, Hsu and his team studied the cytotoxicity of ursolic acid on the A549 lung cancer cell line. Ursolic acid has been found to increase P21 expression via P53 upregulation while deactivating cyclins/CDKs [92]. Concerning lupeol, the antiproliferative effect of this compound on prostate cancer cell lines has been reported. It caused G2/M arrest in PC-3 cells through *β*-catenin signaling suppression, resulting in apoptosis [93,94]. Pomolic and Euscaphic acids are pentacyclic triterpenoids with anticancer properties. The antiproliferative and antiapoptotic effects of pomolic acid against docetaxel-resistant PC-3 prostate cancer cells were proven [95]. Besides this, it caused cell proliferation inhibition in MCF-7 cells, together with the induction of sub-G (1) arrest mediated by the increasing mRNA levels of the two apoptotic genes p53 and p21, together with caspase-3 and -9 activation [96]. Euscaphic acid was found to possess an antiproliferative effect on NPC cancer cells; it arrested the cell cycle in the G1/S phase and induced apoptosis through the deactivation of the PI3K/AKT/mTOR pathway [97]. Finally, the anticancer effect of gallic acid and its methyl ester is known. Recently, gallic acid’s effect on the inhibition of prostate cancer progression in LNCaP and PC-3 prostate cancer cells was inspected. It was found that gallic acid has depleted the mitochondrial membrane potential (ΔΨm) and induced DNA fragmentation and apoptosis. Gallic acid regulates the expression of apoptotic genes, besides down-regulating HDAC1 and 2 expressions, resulting in elevated cetyl-p53 expression, subsequent to the decreased expression of cell-cycle-regulatory genes such as cyclin D1 and E1, and the increased expression of p21, a cell cycle arrest gene [98]. Moreover, gallic acid and its methyl ester prevented NF-κB transcriptional activity, thereby inhibiting the growth of DU145 prostate cancer cells [99]. Finally, it worth mentioning that gallic acid augmented the apoptotic effect of paclitaxel and carboplatin in MCF-7 breast cancer cells through increased Bax and P53 expression [100].

Henceforth, the chemical profiling of *P. curviflorus* using LC-MS/MS analysis could explain the relationship between its metabolites and its demonstrated anticancer activity.

**Table 4:** Metabolites identified in *P. curvifloris* crude extract using LC-ESI/TOF/MS/MS.

	Rt (min)	Measured *m*/*z*	Calculated *m*/*z*	Mass Error (ppm)	Adduct	Molecular Formula	MS/MS Spectrum	Deduced Compound	Ref.
1	1.17	169.0137	169.0137	0	[M − H]^−^	C_7_H_6_O_5_	169, 125	Gallic acid	[101,102]
2	1.35	343.0936	343.1029	−27.1	[M + H]^+^	C_15_H_18_O_9_	343, 325, 283	1-Caffeoyl-*β*-D-glucose	[103]
3	4.61	289.0706	289.0712	−2.07	[M − H]^−^	C_15_H_14_O_6_	289, 245,205, 179	Catechin	[104]
4	4.87	427.1018	427.1029	−2.57	[M + H]^+^	C_22_H_19_O_9_	427,275, 150	Curviflorin	[40]
5	4.90	183.0309	183.0293	8.74	[M − H]^−^	C_8_H_8_O_5_	183, 168, 140, 124	Methyl gallate	[34,102,105]
6	4.95	183.0633	183.0657	−13.11	[M + H]^+^	C_9_H_10_O_4_	183, 168,140, 123	Syringaldehyde	[102,106,107]
7	5.03	153.0572	153.0552	13.07	[M + H]^+^	C_8_H_8_O_3_	153, 135, 125, 93	Vanillin	[102,106,108]
8	6.26	609.1453	609.1456	−0.49	[M − H]^−^	C_27_H_30_O_16_	609,449,301, 300	Rutin	[34,109]
9	6.54	463.0896	463.0871	4.12	[M − 2H]^−^	C_21_H_21_O_12_^+^	463, 301,300,227	Myrtillin	[102,110,111]
10	6.71	611.1911	611.1976	−10.63	[M + H]^+^	C_28_H_34_O_15_	611, 303, 268	Hesperidin	[34,102,109]
11	7.01	443.0958	443.0978	−4.51	[M + H]^+^	C_22_H_18_O_10_	443,425,151, 123	3,3’,4’,5,7-pentahydroxyflavane−5-*O*-gallate	[40,112]
12	7.22	447.0930	447.0927	0.67	[M − H]^−^	C_21_H_20_O_11_	447, 385, 301, 284	Quercetrin	[101,109]
13	7.28	477.1032	477.1033	−0.21	[M − H]^−^	C_22_H_22_O_12_	477, 314, 285, 271, 243	Isorhamnetin−3-glucoside	[102,110,111]
14	7.56	459.0927	459.1054	27.7	[M + H]^+^	C_22_H_19_O_11_	459, 441, 307	3,3’,4’,5,5′,7-hexahydroxyflavane−5-*O*-gallate	[40]
15	9.51	301.0359	301.0348	3.65	[M − H]^−^	C_15_H_9_O_7_	301,284, 255, 151	Quercetin	[34,109]
16	9.94	303.0467	303.0499	−10.56	M ^+^	C_15_H_11_O_7_^+^	303, 284, 274, 257, 247, 229	Delphinidin	[113]
17	9.94	315.0544	315.0505	12.38	[M − H]^−^	C_16_H_12_O_7_	315, 300, 285, 271, 243, 151	Isorhamnetin	[41,114]
18	9.99	285.0390	285.0399	−3.16	[M − H]^−^	C_15_H_10_O_6_	285, 257, 241,223,197, 151	Kaempferol	[41]
19	20.86	599.4351	599.4288	10.51	[M + Na]^+^	C_35_H_60_O_6_	599, 413	*β*-Sitosterol−3-O-*β*-D-glucoside	[115]
20	22.03	487.3445	487.3423	4.51	[M − H]^−^	C_30_H_48_O_5_	487, 425, 279	Euscaphic acid	[116,117]
21	22.73	471.3469	471.3474	−1.06	[M − H]^−^	C_30_H_48_O_4_	471, 453, 409	Pomolic acid	[116]
22	23.03	439.3575	439.3576	−0.23	[M + H^+^ − H_2_O]^+^	C_30_H_48_O_2_	439, 393, 215, 203, 161, 147, 95	Ursolic acid	[118,119]
23	24.54	409.387	409.3834	8.79	[M + H^+^ − H_2_O]^+^	C_30_H_50_O	409, 137, 109	Lupeol	[119,120]
24	25.29	395.3592	395.3678	−21.75	[M + H^+^ − H_2_O]^+^	C_29_H_48_O_1_	395, 378, 311, 297, 255, 161, 147	Stigmasterol	[120,121]
25	26.51	397.3866	397.3834	8.05	[M + H^+^ − H_2_O]^+^	C_29_H_50_O_1_	397, 255, 161, 147	*β*-Sitosterol	[120,121,122]

Cyclin-dependent kinase-2 (CDK-2) and epidermal growth factor receptor (EGFR) are key proteins in the cell signaling pathways controlling its survival and apoptosis, and they could help researchers develop a new target and effective treatment approach for cancer patients. The EGFR tyrosine kinase pathway, and mitochondrial membrane permeability mediated by Bax and Bcl-2, are thought to be involved in p53 and caspase-3 activation [123]. On the other hand, cyclin E/CDK-2 is involved in the G1 and G1-S phases’ transition, and it controls the apoptotic response to DNA damage via FOXO1 protein phosphorylation, which has a crucial role in controlling the cell cycle progression [123]. Furthermore, several chemotherapies can trigger cancer cells’ apoptosis via G1 arrest mediated by CDK-2 downregulation [123,124].

To put further emphasis on the differential anticancer effects of the two test extracts on PC-3 prostate cancer cells, all the phytochemicals—either isolated or identified by LC-MS/MS analysis—in *P. acaciae* [26,34] and *P. curviflorus* [35,36,37,38,39,40] were explored using molecular docking simulations for their binding modes towards both CDK-2 and EGFR proteins. As demonstrated in Table S1, the molecular docking studies indicated that the majority of the investigated compounds—chiefly those of *P. curviflorus*—exhibited noticeable binding affinities towards both proteins, with binding energies of −10.69 to −16.39 Kcal/mol inside CDK-2 protein, and inside EGFR protein from −14.68 to –18.69 Kcal/mol. Additionally, they formed promising interactions with the key amino acids Leu 83 and Lys 89 inside the CDK-2 protein, and Met 769 inside the EGFR active sites. It deserves mention that the docking results in the current study are in line with the reported antitumor activities of these phytoconstituents, especially for prostate cancer, and their cycle arrest and apoptotic effects are mediated through the deactivation of cyclins/CDKs and the activation of apoptotic proteins such as caspases, p53 and p21, the CDK inhibitor [55,56,57,58,59,60,61,62,63,64,65,66,67,68,69,70,71,72,73,74,75,76,77,78,79,80,81,82,83,84,85,86,87,88,89,90,91,92].

**Figure 9:**
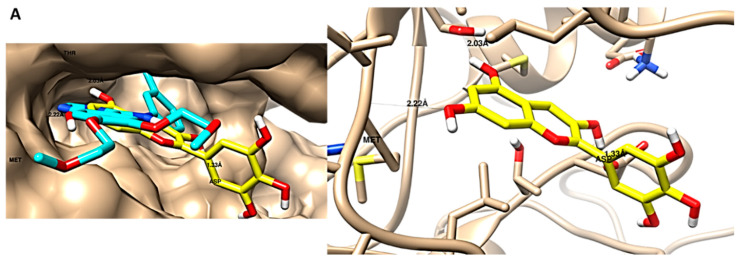

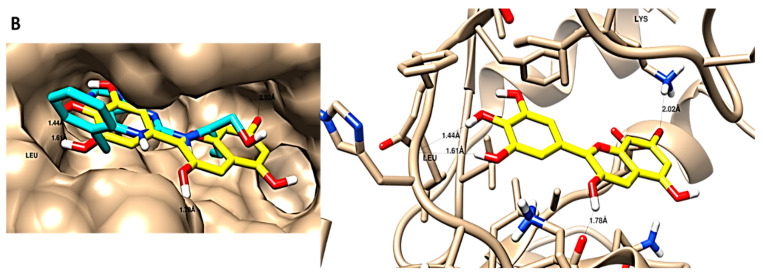
Ligand disposition and ligand–receptor interactions of delphinidin (the compound with the least binding energy) inside the EGFR protein (**A**) and CDK-2 protein (**B**). The right panel represents the interactive mode, and the left panel demonstrates a surface representation. Using Chimera software, the 3D images were made.

Fortunately, ten compounds exhibited the mutual inhibition of both targets in our study: CDK-2 and EGFR. Their binding disposition and interconnections with the key amino acids were quite close to the co-crystallized ligand. These phytochemicals were epicatechin, catechin, gallic acid, methyl gallate, delphinidin, isorhamnetin, isorhamnetin-3-*O*-glucoside, *β*-sitosterol glucoside, euscaphic acid and curviflorin. Among these, catechin, gallic acid and methyl gallate were identified in both plants, epicatechin was reported only in *P. acacia*, and the other compounds were exclusively present in *P. curviflorus*. Figure 9 shows the ligand disposition and ligand–receptor interactions of delphinidin inside the EGFR and CDK-2 proteins, since it displayed the least binging energy among the tested compounds towards the two targets. Thus, these findings could give an insight into the pronounced anticancer effect of *P. curviflorus* compared to *P. acacia.*

## 4. Conclusions

Herein, extracts of *P. acacia* and *P. curviflorus* were assessed for their cytotoxicity on a panel of cancerous cells, along with their antioxidant potential and total phenolic contents (TPC). *P. curviflorus* exhibited higher TPC, antioxidant effects and antiproliferative and apoptotic activities on PC-3 cancer cells compared to *P. acacia*, and seemed to be a promising chemopreventive and anticancer herb, thanks to its phytochemicals—more precisely, its phenolics. Therefore, further advance studies are needed in order to validate the advantages of *P. curviflorus* for the alleviation of prostate malignancies in humans.

## Figures and Tables

**Table 1:** List of the sequences in forward and reverse for the tested genes.

Gene	Forward	Reverse
P53	5′-CCCCTCCTGGCCCCTGTCATCTTC-3′	5′-GCAGCGCCTCACAACCTCCGTCAT-3′
BAX	5′-GTTTCATCCAGGATCGAGCAG-3′	5′-CATCTTCTTCCAGATGGTGA-3′
CASP-3	5′-TGGCCCTGAAATACGAAGTC-3′	5′-GGCAGTAGTCGACTCTGAAG-3′
CASP-8	5′-AATGTTGGAGGAAAGCAAT-3′	5′-CATAGTCGTTGATTATCTTCAGC-3′
CASP-9	5′-CGAACTAACAGGCAAGCAGC-3′	5′-ACCTCACCAAATCCTCCAGAAC-3′
BCL2	5′-CCTGTGGATGACTGAGTACC-3′	5′-GAGACAGCCAGGAGAAATCA-3′
β-actin	5′-GTGACATCCACACCCAGAGG-3′	5′-ACAGGATGTCAAAACTGCCC-3′

**Table 2:** Comparison of the antioxidant activities of *P. acacia* and *P. curviflorus* by DPPH, FRAP and TAC assays.

Sample	DPPH (IC_50_ in µg/mL)	FRAP (mMol Fe^+2^/g)	TAC (mg GAE/g)
*P. acacia*	60.70 ± 4.28 ^a^	1.29 ± 0.31 ^c^	32.67 ± 2.81 ^c^
*P. curviflorus*	48.28 ± 3.41 ^b^	1.43 ± 0.54 ^c^	41.89 ± 3.15 ^b^
Ascorbic acid	10.64 ± 0.82 ^c^	3.14 ± 0.82 ^b^	69.23 ± 4.51 ^a^
BHT	-	8.07 ± 0.79 ^a^	-
ANOVA (*p*-value)	<0.001 ***	<0.001 ***	<0.001 ***

*** Significant at *p* < 0.001. Means followed by different letters (^a,b,c^) are significantly different according to DMRTs.

**Table 3:** Cytotoxic activity of the two tested extracts against a panel of cancerous cells, measured through the application of the MTT assay.

Sample	IC_50_ (μg/mL)			
	Prostate	Breast	Ovarian	Lung
	PC-3	MDA-MB-231	A2780	A549
***P. acacia***	34.12 ± 1.3 ^a^	86.5 ± 2.01	NA	50.6 ± 1.63
***P. curviflorus***	25.83 ± 1.2 ^b^	NA	76.5 ± 1.78	NA
**Doxorubicin**	8.23 ± 0.56 ^c^	7.07 ± 0.64	10.63 ± 0.76	9.26 ± 0.64
ANOVA (*p*-value)	<0.001 ***^F^	<0.001 ***^T^	<0.001 ***^T^	<0.001 ***^T^

NA = Not active. The data were obtained as the Mean ± SD of three independent values. The IC_50_ (μg/mL) values were estimated using GraphPad Prism 7 software (Dotmatics, San Diego, CA, USA). ^F^; One way ANOVA, ^T^; independent *t*-test, ^a,b,c^ means followed by different letters are significantly different according to DMRTs at 0.05 level. *** Significantly different at *p* < 0.001 according to an independent sample *t*-test.

## Data Availability

Data is contained within the article and Supplementary Materials.

## Supplementary Materials

The following supporting information can be downloaded at https://www.mdpi.com/article/10.3390/antiox11071249/s1. Table S1: Summary of the ligand–receptor interactions of the identified docked compounds in both *P. acaciae* and *P. curviflorus* extracts towards cyclin-dependent kinase (CDK2) and epidermal growth factor receptor (EGFR) binding sites. Figure S1: Total ion chromatogram (TIC) recorded in the negative mode for *P. curviflorus* extract. Figure S2: Total ion chromatogram (TIC) recorded in the positive mode for *P. curviflorus* extract.
