# Supplementary material for: Comparative Assessment of the Antioxidant and Anticancer Activities of Plicosepalus acacia and Plicosepalus curviflorus: Metabolomic Profiling and In Silico Studies
Source: Antioxidants (Basel). 2022 Jun 25;11(7):1249. doi: 10.3390/antiox11071249 (PMC9311546; doi:10.3390/antiox11071249)
Supplement: Supplementary file 1 [file antioxidants-11-01249-s001.zip › antioxidants-1748089-supplementary.pdf]

antioxidants

MDPI

Article

Comparative Assessment of the Antioxidant and Anticancer Activities of *Plicosepalus acacia* and *Plicosepalus curviflorus*: Metabolomic Profiling and I*n Silico* Studies.

Additional Experimental Detail

1. **Table S1.** Summary of ligand-receptor interactions of the identified docked compounds in both *P. Acaciae* and *P. Curviflorus* extracts towards cyclin-dependent kinase (CDK2) and Epidermal growth factor receptor (EGFR) binding sites.
2. **Figure S1.** Total ion chromatogram (TIC) recorded in the negative mode for *P. curviflorus* extract.
3. **Figure S2.** Total ion chromatogram (TIC) recorded in the positive mode for *P. curviflorus* extract.

Table S1. Summary of ligand-receptor interactions of the identified docked compounds in both *P. Acaciae* and *P. Curviflorus* extracts towards cyclin-dependent kinase (CDK2) and Epidermal growth factor receptor (EGFR) binding sites.

	Compound	Ligand-receptor interactions towards CDK-2 (PDB=2a4l)*	Ligand-receptor interactions towards EGFR (PDB=1M17)#
	**Co-crystallized ligands (Key interactions)**	**2 HB with Leu 83<br>1 arene-cation interaction with Lys 89**	**1 HB with Met 769**
P. Acacia	Catechin-7-O gallate	-	-
	Quercetin-3-O-β-(6-O-galloyl)-gluocopyranoside	1 arene-cation interaction with Lys 89	1 HB with Met 769
	Loranthin	1 arene-cation interaction with Lys 89	-
	Betulinic acid	-	-
	Taxifolin	-	-
	Datiscin	1 arene-cation interaction with Lys 89	1 HB with Met 769
	Naringenin	1 arene-cation interaction with Lys 89	-
	Apigenin	1 arene-cation interaction with Lys 89	-
	Hesperetin	1 arene-cation interaction with Lys 89	1 HB with Met 769
	Epicatechin	1 HB with Leu 83 + 1 arene-cation interaction with Lys 89	1 HB with Met 769
	Vanillic acid	1 HB with Leu 83	-
	Diosmetin	1 arene-cation interaction with Lys 89	-
P.	Myrtillin	1 HB with Leu 83 + 1 arene-cation interaction with Lys 89	-
	Delphinidin	1 HB with Leu 83 + 1 arene-cation interaction with Lys 89	1 HB with Met 769
	Isorhamnetin	1 HB with Leu 83 + 1 arene-cation interaction with Lys 89	1 HB with Met 769
	Kaempferol	-	-
	Quercitrin	1 arene-cation interaction with Lys 89	-

	Isorhamnetin -3- *O*- glucoside	1 HB with Leu 83 + 1 arene-cation interaction with Lys 89	1 HB with Met 769
	Syringaldehyde	-	-
	Vanillin	-	-
	β- sitosterol 3-*O*-*β* D glucoside	1 HB with Leu 83 + 1 arene-cation interaction with Lys 89	1 HB with Met 769
	Stigmasterol	1 arene-cation interaction with Lys 89	--
	Euscaphic acid	1 HB with Leu 83 + 1 arene-cation interaction with Lys 89	1 HB with Met 769
	Pomolic acid	1 HB with Leu 83	1 HB with Met 769
	Lupeol	1 HB with Leu 83	-
	Ursolic acid	1 arene-cation interaction with Lys 89	1 HB with Met 769
	Chlorogenic acid	1 arene-cation interaction with Lys 89	1 HB with Met 769
	1-Caffeoyl-*β*-D-glucose	1 HB with Leu 83	1 HB with Met 769
	3,3' ,4' ,5,7-pentahydroxyflavane-5-*O*-gallate	1 HB with Leu 83	1 HB with Met 769
	3,3' ,4' ,5,5′,7-hexahydroxyflavane-5-*O*-gallate	1 HB with Leu 83 + 1 arene-cation interaction with Lys 89	-
	Plicosepalin [(+) catechin-4′-*O*-(1′′-O-galloyl-5′′-*O*-methyl)- myo-inositol]	1 HB with Leu 83	-
	4- methoxy cinnamic acid	-	-
	2S,3R-3,3′,4′,5,5′,7-hexahydroxyflavane-3′,5-di-*O*-gallate	1 arene-cation interaction with Lys 89	1 HB with Met 769
	Curviflorin	1 HB with Leu 83 + 1 arene-cation interaction with Lys 89	1 HB with Met 769
	Curviflorside	1 arene-cation interaction with Lys 89	1 HB with Met 769
	Pentahydroxy flavan di-*O*-gallate-mono protocatechuate	1 arene-cation interaction with Lys 89	1 HB with Met 769
**Compounds present in the two**	Catechin	1 HB with Leu 83 + 1 arene-cation interaction with Lys 89	1 HB with Met 769
	Quercetin	1 arene-cation interaction with Lys 89	-
	Quercetin-3-*O*-*β*-D-gluocopyranoside	1 HB with Lys 89 + 1 arene-cation interaction with Lys 89	-
	Rutin	1 HB with Lys 89	-
	Hesperidin	1 arene-cation interaction with Lys 89	1 HB with Met 769
	β-Sitosterol	-	1 HB with Met 769
	Gallic acid	1 HB with Leu 83 + 1 arene-cation interaction with Lys 89	1 HB with Met 769
	Methyl gallate	HB with Leu 83 + 1 arene-cation interaction with Lys 89	1 HB with Met 769
	Caffeic acid	1 arene-cation interaction with Lys 89	1 HB with Met 769
	Protocatechuic acid	1 HB with Leu 83	-

* The binding energies of the docked compounds inside CDK-2 protein from -10.69 to -16.39 Kcal/mol, #. The binding energies of the docked compounds inside EGFR protein from -14.68 to –18.69 Kcal/mol.

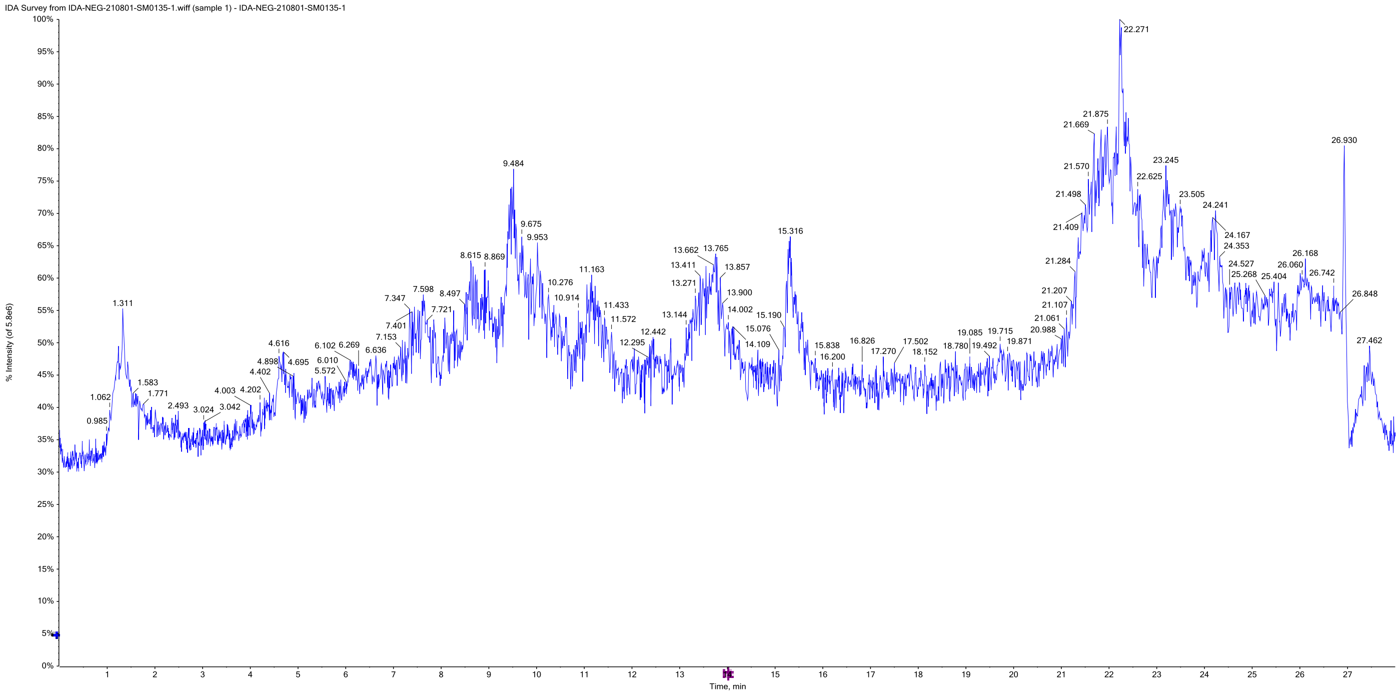

Figure S1. Total ion chromatogram (TIC) recorded in the negative mode for *P. curviflorus* extract.

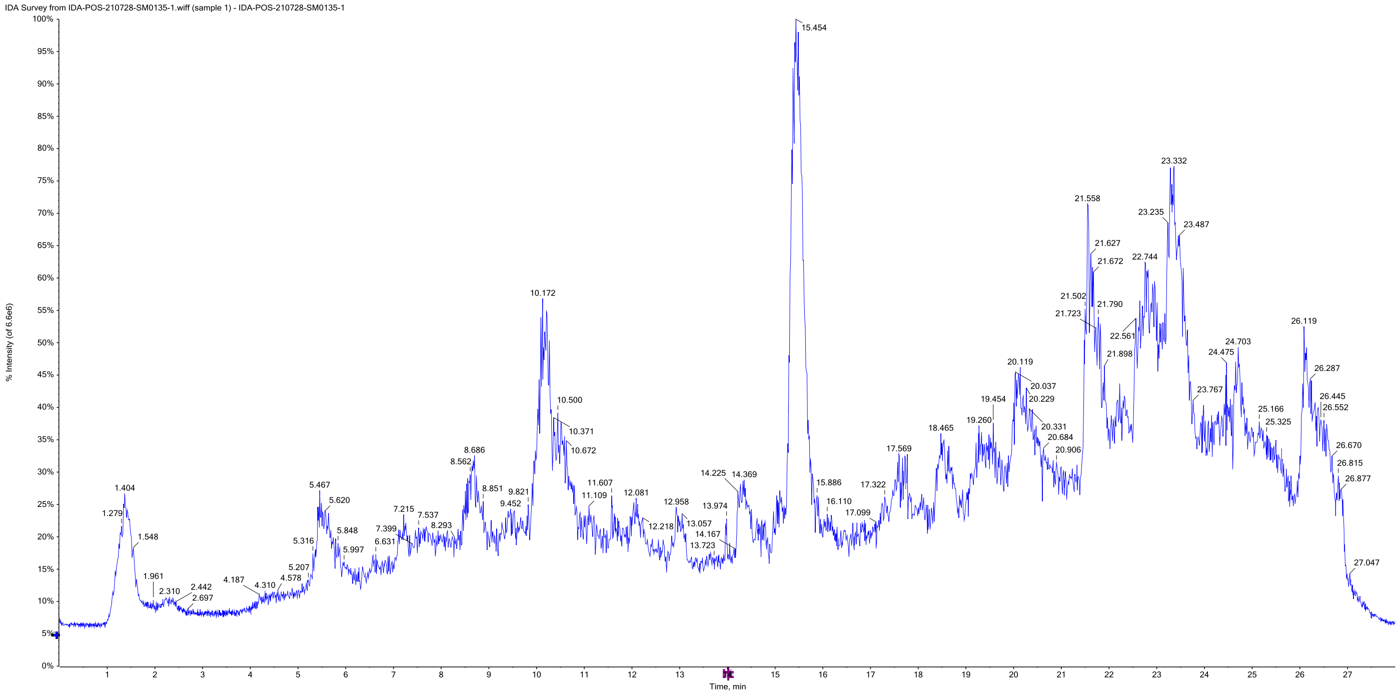

Figure S2. Total ion chromatogram (TIC) recorded in the positive mode for *P. curviflorus* extract.
